# Prevalence, prenatal screening and neonatal features in children with Down syndrome: a registry- based national study

**DOI:** 10.1186/s13052-015-0192-9

**Published:** 2015-10-28

**Authors:** Tatjana Glivetic, Urelija Rodin, Milan Milosevic, Diana Mayer, Boris Filipovic-Grcic, Maida Seferovic Saric

**Affiliations:** General Hospital Zabok, Bracak 6, Zabok, Croatia; University of Zagreb, School of Medicine, A Stampar Schoolof Public health and Croatian National Institute of Public Health, Rockefellerova 7, Zagreb, Croatia; University of Zagreb, School of Medicine, A Stampar Schoolof Public health, Rockefellerova 4, Zagreb, Croatia; Croatian National Institute of Public Health, Rockefellerova 7, Zagreb, Croatia; University Hospital Centre Zagreb, Kispaticeva 12, Zagreb, Croatia

**Keywords:** Down syndrome, Trisomy 21, Prevalence, Prenatal screening, Register

## Abstract

**Background:**

Down syndrome (DS) is one of the most common chromosomal abnormalities among newborns. In recent years advances in perinatal and neonatal care have improved chance of survival for the children with DS. The objective of this Registry-Based study was to get more accurate data of DS prevalence with evaluation of antenatal screening, neonatal and maternal features among total births in Croatia from 2009 to 2012.

**Methods:**

We used retrospectively collected data for DS newborns from the medical birth database and perinatal mortality database for the period of 2009–2012. Differences between DS and the referent population for each year in quantitative measures were assessed with the independent t-test. Other differences in nominal and categorical values were analyzed with the chi-square test.

**Results:**

The total prevalence for DS in the period of 2009–2012 was 7.01 per 10,000 births, while the live-birth prevalence was 6.49 per 10,000 births. The significant differences (*p* < 0.05) between the DS and reference populations for each year were noticed for birth weight and length, gestational age, mother age, Apgar score of ≥6 after 5 min and breastfeeding. Among newborns with DS, there were 64 (53.33 %) males and 56 (46.67 %) females versus 88,587 (51.76 %) males and 82,553 (48.23 %) females in the reference population. In the DS group compared to the reference population the mean birth weight was 2845 grams versus 3467 grams in males and 2834 grams versus 3329 grams in females, respectively, with a mean birth length of 47 cm versus 50 cm for both genders. The mean gestational age of the DS births was 37 weeks and the mean age of the mothers was 32.6 years, versus 39 weeks and 29.1 years, respectively, in the reference population. Only 68.3 % of children with DS were breastfed from birth, compared with 94.72 % of children in the reference population.

**Conclusions:**

The significant differences for neonatal and maternal features between DS and the referent population were found similar to other studies. The total prevalence of DS in Croatia in the period of 2009–2012 was lower than the previously estimated prevalence based on EUROCAT data. The establishment of a new national registry of congenital malformations covering 99 % of all births in Croatia is necessary to improve the health and prosperity of children, adolescents and adults with DS in Croatia.

## Background

Down syndrome (DS) is one of the most common chromosomal abnormalities among newborns. DS remains the most easily recognized condition and is associated with increased risk for cardiologic, endocrinologic, hematologic and respiratory diseases. In recent years, advances in perinatal and neonatal care have improved the chances of survival in children with DS [1].

EUROCAT (European Surveillance of Congenital Anomalies) is the principal source of information on the epidemiology of congenital anomalies in Europe. It currently surveys more than 1.7 million births per year, covered by 43 registries in 23 countries [2].

In Europe, the total prevalence of DS is 22.0 per 10,000 births (11.2 per 10,000 live births) while the estimated prevalence in Croatia is 12.35 per 10,000 births (10.54 per 10,000 live births) [3, 4].

In Croatia, the registry that provides data for EUROCAT is population-based, covering only 20.8 % of total births. The registry covers northwestern Croatia in only urban areas, two at the seaside (Pula, Rijeka) and another two from continental areas (Varaždin, Koprivnica). In order to get more accurate data regarding DS prevalence and perinatal mortality at the national level, as well as on the neonatal and maternal features of DS newborns, the total births from 2009 to 2012 in Croatia were evaluated. The medical birth database used in this study covers all births at health institutions and includes all mothers and newborns with pathological conditions.

## Methods

The data for this study were obtained from all health institutions for the period of 2009–2012. Data were processed at the Croatian National Institute of Public Health. In Croatia, new birth certificates and perinatal death certificates have been introduced in routine health statistics since 2000 in accordance with World Health Organisation (WHO) recommendations, by using features from the Obstetrical Quality Indicators and Data collection (OBSQID) basic information sheet for the recording of births [5]. Perinatal deaths have been collected through perinatal death certificates and coded according to the International Classification of Diseases (ICD-10 revision) [6]. The birth form includes a significant number of core and recommended EURO-PERISTAT indicators, proposed by the PERISTAT scientific committee. The mother’s and the newborn’s pathologic conditions are recorded according to ICD-10 revision codes [6]. In the present study, all records with ICD-10 codes were included (Q90: trisomy 21; Q90.0: meiotic nondisjunction of chromosome 21; Q90.1: mosaic Down syndrome; Q90.2: translocations involving chromosome 21; Q90.9: Down syndrome, unspecified). Comparisons between DS and a reference population were done for timing of the first antenatal visit and ultrasound examinations during pregnancy, birth weight and length, gestational age, maternal age at delivery, Apgar score of ≥6 after 5 min, breastfeeding after birth, pathology/complications in the newborn, educational level of the mother, smoking, and consumption of alcohol or psychoactive drugs during pregnancy.

Differences between DS and the reference population for each year in quantitative measures (birth weight, birth length, gestational age and mother age) were assessed with the independent t-test. Other differences in nominal and categorical values, including differences in total prevalence of DS per 10,000 births, were analyzed with the chi-square test. All *p-*values below 0.05 were considered significant. The StatsDirectversion 3.0.86 statistical software was used in all statistical procedures [7].

## Results

In the period from 2009 to 2012, there were 171,140 total births in Croatia. Among them, a total of 120 children were born with DS, confirmed by chromosomal analysis. The major chromosomal aberrations were trisomy 21 (Q90, Q90.9) in 76.6 % (*n* = 92) of the DS newborns and meiotic nondisjunction of chromosome 21 (Q90.0) in 20.8 % (*n* = 25), while the remaining cases were caused by mosaicism-mitotic nondisjunction (Q90.1).

**Fig. 1:**
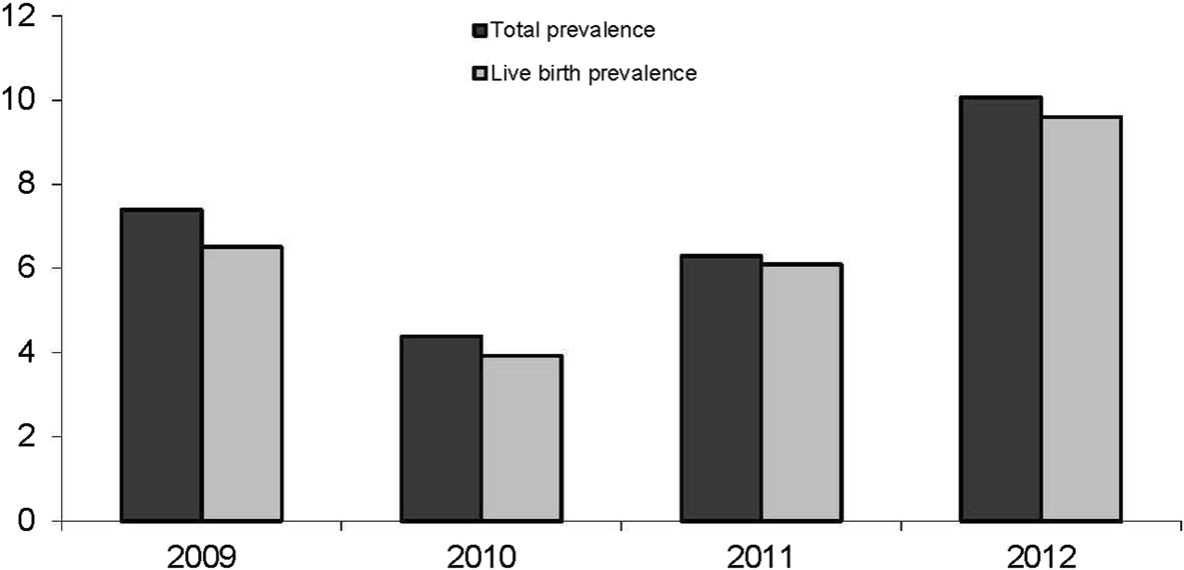
Total and live birth prevalence of DS per 10 000 births for period from 2009 to 2012 in Croatia

Figure 1 shows the total and live-birth prevalence for the observed period. The DS prevalence ranged from 4.38 to 10.06 per 10,000 births. A similar trend was noticed for live-birth prevalence. The total prevalence in this period was 7.01 per 10,000 births, while the live-birth rate was 6.49 per 10,000 births.

Fourteen perinatal deaths (11.7 % of those born with DS) were recorded in the period of 2009–2012. In this period, the perinatal mortality rate for DS was 1,203.8 per 10,000 total births and for the reference population was 68.4 per 10,000 total births, indicating at least a 17-times higher perinatal mortality for DS compared to the reference population (Table 1). Average fetal mortality caused by DS was 709.25 per 10,000 total births, while early neonatal mortality was 528 per 10,000 live births. For the reference population, fetal and neonatal mortality was also 15–20 times lower at 42.1 and 25.8, respectively.

**Table 1:** Characteristic of children with Down syndrome (DS) and the reference population (REF)

	2009.		2010.		2011.		2012.	
	DS	REF	DS	REF	DS	REF	DS	REF
	*N* = 33	*N* = 44673	*N* = 19	*N* = 43400	*N* = 26	*N* = 41306	*N* = 42	*N* = 41761
Birth weight (g, mean)	2906.7^a^	3409.8	2954.2^a^	3428.6	2954.2^a^	3389.4	2763.2^a^	3396.7
Birth length (cm, mean)	47.4^a^	50.2	46.1^a^	50.3	47.5^a^	50.1	46.9^a^	50.1
Gestational age (weeks, mean)^b^	36.7^a^	39.1	36.7^a^	39.2	37.8^a^	39.0	37.0^a^	39.0
Apgar ≥ 6 after 5 min^b^	22 (66.7 %)^a^	39694 (99.2 %)	12 (63.1 %)^a^	37728 (98.9 %)	20 (76.9 %)^a^	36079 (99.0 %)	30 (71.4 %)^a^	36859 (99.3 %)
Intervention on delivery	6 (18.2 %)	8913 (19.9 %)	7 (36.8 %)	9224 (21.3 %)	7 (26.9 %)	7213 (17.5 %)	19 (45.2 %)	8845 (21.2 %)
Parity 1	6 (18.2 %)	15002 (33.6 %)	8 (42.1 %)	14776 (34.0 %)	8 (30.8 %)	14083 (34.1 %)	12 (28.6 %)	14359 (34.4 %)
Parity 2	6 (18.2 %)	5643 (12.6 %)	1 (5.3 %)	5289 (12.2 %)	5 (19.2 %)	5015 (12.1 %)	5 (11.9 %)	5116 (12.3 %)
Parity 3	3 (9.1 %)	1597 (3.6 %)	1 (5.3 %)	1525 (3.5 %)	3 (11.6 %)	1469 (3.6 %)	3 (7.1 %)	1483 (3.6 %))
Parity ≥4	3 (9.1 %)	1017 (2.3 %)	0 (0.0 %)	1044 (2.4 %)	1 (3.8 %)	1015 (2.5 %)	1 (2.4 %)	1024 (2.5 %)
Parity 0	15 (45.4 %)	21414 (47.9 %)	9 (47.4 %)	20766 (47.8 %)	9 (34.6 %)	19724 (47.8 %)	21 (50.0 %)	19779 (47.4 %)
Mother age (years, mean)	32.7^a^	29.0	31.8^a^	28.2	32.0^a^	30.0	33.2^a^	29.2
Breast feeding	21 (63.6 %)^a^	42676 (95.5 %)	11 (57.9 %)^a^	40812 (94.0 %)	19 (73.1 %)^a^	39413 (95.4 %)	31 (73.80 %)^a^	40268 (96.4 %)
Perinatal mortality (on 10000)	5 (1515.2)	343 (76.8)	3 (1578.9)	323 (74.4)	2 (769.2)	268 (64.9)	4 (952.4)	240 (57.5)
Fetal mortality (on 10000)	4 (1212.1)	191 (42.7)	1 (526.3)	210 (48.4)	1 (384.6)	158 (38.3)	3 (714.3)	162 (38.8)
Early neonatal mortality (on 10000 newborns)	1 (344.8)	152 (34.2)	2 (1111.1)	111 (25.7)	1 (400.0)	102 (24.8)	1 (256.4)	77 (18.5)

^a^Significant differences (*P* < 0.05) between DS regarding referent population for each year (birth weight, birth length, gestational age and mother age were assessed with independent t-test; other differences with chi-square test)

^b^For Apgar scoring total N for referent population were 40022 (2009), 38118 (2010), 36444 (2011) and 37119 (2012)

Among newborns with DS, there were 64 (53.33 %) males and 56 (46.67 %) females versus 88,587 (51.76 %) males and 82,553 (48.23 %) females in the reference population. In the DS group compared to the reference population, the mean birth weight was 2845 grams versus 3467 grams in males and 2834 grams versus 3329 grams in females, respectively, with a mean birth length of 47 cm versus 50 cm for both genders. The mean gestational age of the DS births was 37 weeks and the mean age of the mothers was 32.6 years, versus 39 weeks and 29.1 years, respectively, in the reference population. Of the DS births, there were 80 (66.7 %) pregnancies in women aged ≥35 years. Only 68.3 % of children with DS were breastfed from birth, compared with 94.72 % of children in the reference population. Additionally, significant differences (*p* < 0.05) between the DS and reference populations for each year were noticed for birth weight and length, gestational age, mother age, Apgar score of ≥6 after 5 min and breastfeeding, while there were no significant differences in parity (Table 1).

In 7 of the DS newborns, there were one or more additional structural anomalies (Table 2). In 6 (5 %) of the DS newborns, congenital heart defects (CHDs) were detected, the most common being atrial septal defects (*n* = 3, 2.5 %), followed by one newborn with a ventricular septal defect, an atrioventricular septal defect and persistent ductus Botalli. Also, in one newborn, anomalies of the urinary system were detected. Other additional conditions included 8 % (*n* = 10) of newborns with perinatal asphyxia, 0.8 % (*n* = 1) with thrombocytopenia, and 0.8 % (*n* = 1) with leukemia.

**Table 2:** Comorbidity of children with Down syndrom

Comorbidity of children with Down syndrom, *n* = 120	
Congenital heart defects	5 % (*n* = 6)
Anomalies of the urinary system	0.8 % (*n* = 1)
Perinatal asphyxia	8 % (*n* = 10)
Trombocytopenia	0.8 % (*n* = 1)
Leukemia	0.8 % (*n* = 1)
Total	15 % (*n* = 19)

Unfortunately, gestational age at first antenatal visit was available only for 85 (70.8 %) of the children with DS. Among them, in 79 cases of DS, the first antenatal visit occurred in the first 14 weeks of gestation, which meets the recommendations of the National Health Care Program. Similarly, the first ultrasound examination should be performed by 14 weeks of gestation, and our data point out that the first ultrasound examination was mainly done at the same time as the first antenatal visit. In addition, anomaly-scanning ultrasounds were recorded for 46 children with DS. The Croatian National Health Care Program recommends structural-anomaly scanning at 18–22 weeks of gestation, which was performed only in 38.3 % of the DS pregnancies.

Furthermore, the collected social features did not show obvious risk behaviours in the mothers. For example, of the total of 120 mothers, 91.7 % declared themselves non-smokers and 95 % denied using alcohol or psychoactive drugs during pregnancy. Also, there were no similarities in the mothers’ levels of education. According to available data, most had a secondary-school education followed by college/university degree, and three had only a primary-school education.

## Discussion

Congenital anomalies, including DS, present an important public health issue since the surviving children have special medical, social and educational needs. National prenatal screening programs for DS and clinical guidelines for the further care of DS newborns should exist. To create an efficient strategy, population-based data are necessary. The Croatian registry of congenital anomalies was established in 1983 and is limited to the northwest of Croatia, part of central Croatia and part of the Croatian coast (Primorje). This registry covers only 20.8 % of all births and provides data about DC prevalence for EUROCAT. In order to get more reliable data for our study, the prevalence and neonatal characteristics of children with DS were based on total births at health institutions, covering 99 % of all births in Croatia. In this study, for example, 51.7 % of all newborns with DS were born in three large counties that are not included in the Croatian registry of congenital anomalies [8]. The total prevalence of DS in Croatia in the period of 2009–2012 (7.01 per 10,000 births) was lower than the previously estimated prevalence based on EUROCAT data [4]. In the period of 2008–2012, the total prevalence, including terminated pregnancy for fetal anomaly (TOPFA), live births and fetal deaths obtained from EUROCAT were12.96, 8.93 and 8.90 per 10,000, respectively. According to EUROCAT, the total prevalence in Europe was also much higher (22 per 10,000 births) [3]. There are large differences in DS prevalence rates (5.99–43.03), which depend on sociocultural variables and abortion-legislation practices among EU countries. In the period of 2008–2012, TOPFA was performed in 52.3 % of cases of prenatal DS detection (7,335 of a total of 14,036 DS cases). In countries in which abortion is illegal, such as Ireland and Malta, the DS prevalence is higher, varying from 20 to 34 per 10,000 births [4]. In contrast, the DS prevalence in France is quite low (7.5 per 10,000), which is probably due to the high percentage (77 %) of DS pregnancy terminations [9]. In Croatia, TOPFA is legal until 22 weeks of gestation. However, we do not have information on prenatally detected DS, which could result in pregnancy termination and reduced overall prevalence of DS. After 22 weeks of gestation, it is possible to perform TOPFA outside of Croatia in countries with less-restrictive abortion legislation. Also, TOPFA data are not part of routine health statistics; only data about legally induced abortions, spontaneous abortions and pregnancy termination for all fetal anomalies are collected, which does not allow us to make conclusions about pregnancy terminations due to DS. Furthermore, it is important to emphasize that birth records from routine health statistics could be missing data about newborns’ pathologic conditions, which does not allow complete insight into DS prevalence.

EUROCAT has recently analyzed trends in DS prevalence for the period from 1990 to 2009, during which an increasing DS prevalence was noticed. The proportion of births in Europe in the population of mothers aged ≥35 years increased from 13 % in 1990 to 19 % in 2009, which is probably the reason for the increased total prevalence of DS over time [3]. In our study, we found a proportion of 33.3 % of mothers aged ≥35 years who delivered newborns with DS in Croatia. This is very similar to other European countries [3, 9, 10].

Since 1990, widening of and improvements in antenatal screening techniques have resulted in a relatively stable live-birth prevalence in Europe over time [11, 12]. One of the other probable reasons for the stable live-birth prevalence is the availability of prenatal care and routine prenatal ultrasounds. In Croatia, in accordance with national compulsory health insurance, a program of health care measures is recommended. The program includes one general and gynaecologic health examination, with two pregnancy controls in the first trimester, three pregnancy controls in the second trimester, and four pregnancy controls in the third trimester of pregnancy. In pregnancies with complications, the number of visits and diagnostic procedures depends on specialist assessments. In the framework of the program, the national recommendation is to perform three ultrasound scans during a normal pregnancy. The first scan is at 10–14 gestational weeks, followed by routine structural-anomaly scanning at 18–23 weeks, with the last scan at 34–37 weeks. Since 2006, first-trimester screening with biochemical markers and nuchal translucency measurements have been also introduced as optional. In the available records, 92.94 % of DS pregnancies had the first antenatal visit in the first 14 weeks of gestation, which meets the recommendations of the program. In addition, the first ultrasound examination was usually done at the same time as the first antenatal visit. According to a recent report by the Croatian Institute of Public Health, more than 70 % of pregnant women had first-trimester ultrasound screening after 12 weeks of gestation [8]. In most European countries, country-wide policies exist for routine anomaly-scanning ultrasounds. Prenatal screening for DS in many countries has led to large proportions of terminated pregnancies after prenatal diagnosis, which also influences the stable live-birth prevalence over time [13]. For example, according to the United Kingdom’s National Institute for Health and Clinical Excellence (NICE) guidelines, pregnant women in England and Wales should be offered screening for DS that should be performed by the end of the first trimester, no later than at 20 weeks of gestation. The combined test (biochemical markers and nuchal translucency measurement) should be offered between 11 and 14 weeks of gestation, and no later than 20 weeks [14]. According to EUROCAT, in 14 European countries, 66 % of DS cases are detected prenatally and 88 % of those resulted in termination of pregnancy [15]. Detection of fetal anomalies on antenatal ultrasound offers women and their partners information that may help them to better prepare for the birth of their child, including the option of delivery in a setting that permits rapid access to specialists and to surgical or medical care. In our study, we had no information about whether the DS was prenatally or postnatally diagnosed, but in 38 % (46 cases of a total of 120 DS pregnancies), structural anomalies were reported on scanning. The absence of a national screening policy is one of the reasons for the lack of prenatal DS detection data in Croatia. In previous studies, it was observed that in countries with national screening policies, there are measurable impacts on prenatal DS detection rates [13]. The detection rate was higher in countries with primarily first-trimester screening than in those with a mixed first- or second-trimester screening policy. Also, countries with no national screening policies had significantly lower prenatal detection rates for DS [13, 16].

In general, a higher level of education and having previous information on available screening tests, such as a nuchal translucency scan, biochemical tests and invasive diagnostic testing by chorionic villus sampling or amniocentesis, influence a woman’s decisions about which tests to perform [17]. Additionally, the knowledge level about first-trimester DS screening has been positively associated with length of education [18]. In our study, such conclusions could not be made. In the available data, most of the mothers had a secondary-school education followed by a college/university degree, and only three had a primary-school education. However, providing information about available screening options to all pregnant women at the local and national levels is a technique for early and frequent prenatal diagnosis of DS.

In our study, growth retardation in children with DS was found, similar to other results [19, 20]. Previously published charts for DS are based on American, Sicilian, Swedish and Dutch populations, though the American growth charts are most frequently used all over the world [21–24]. Due to significant differences among populations, it is important to have specific DS growth charts for our population, which could be incorporated into our future National Health Care Program for DS children. Also, DS newborns have more frequent cases of birth asphyxia (Apgar score of ≤6 after 5 min) which corresponds to recent literature (2–8 %) [19, 25, 26]. Consequently, newborns with DS have more complications at birth.

Furthermore, 7 of the DS newborns in this study had one or more additional structural anomalies, including 6 with CHD, corresponding to only 6 % of the children. In recent studies, the prevalence of CHD in DS children was much higher, ranging from 43 % to 57 % [25–28]. This low percentage of CHD could be partially explained by the lower number of diagnosed CHDs in our maternity wards. Several authors of studies of DS children with major cardiac malformations found no clinical signs in the first week of life, and therefore a normal neonatal examination does not exclude CHD [29]. If possible, due to these findings, newborns with DS should have echocardiography performed during the first month of life. Additionally, we had records from maternity wards, without further follow-up during the newborn and infant period, and those who were later diagnosed remain unrecorded. A follow-up system should be incorporated into national health policy to provide more accurate data on structural anomalies. Because of the high incidence of a significant CHD, early recognition can lead to the successful early surgical treatment of CHD in children with DS [30].

DS newborns are less frequently breastfed compared with healthy children. The ability to breastfeed may be influenced by a range of difficulties in the first few days of life, often as a consequence of facial and other anatomical structural abnormalities associated with DS. Also, severe neonatal illness is common among DS newborns, leading to hospital admissions that are usually associated with medical interventions and mother-infant separations that can interfere with breastfeeding. In our study, we found similar results, with 68.3 % of children with DS being breastfed from birth, compared with 94.72 % in the reference population. Our percentage of breastfed DS children was higher than that reported in some studies (43 %–48 %), suggesting that possible improvements, through the training of maternity-ward health professionals and home-based support of breastfeeding, could be made [19, 31].

## Conclusion

Our study, for the first time, investigates the DS prevalence and neonatal characteristics among all newborns in Croatia. The significant differences for neonatal and maternal features between DS and the referent population were found similar to other studies. The total prevalence of DS in Croatia in the period of 2009–2012 was lower than the previously estimated prevalence based on EUROCAT data. Additionally, we provided better insight into some biological and social features that influence perinatal outcomes, which could also be useful in developing preventive health care measures. Through this study, the need to have a national screening policy for fetal congenital anomalies was once again pointed out. Such a policy should involve mandatory anomaly-scanning for all pregnant women, which would increase the opportunities to discover fetal congenital anomalies and would help to prepare families to cope with them. The establishment of a new national registry of congenital malformations covering 99 % of all births in Croatia would be the best way to obtain the relevant information, which is not always possible to find through routine health statistics. Without population-based data about DS prevalence, preventive health care programs will not be sufficiently effective. Also, a plan for age-appropriate health care should be created to improve the health and prosperity of children, adolescents and adults with DS in Croatia.
